# Exploring the ambivalence model of suicidality among Iranian patients: a qualitative study with suicide attempters

**DOI:** 10.3389/fpsyt.2026.1852185

**Published:** 2026-07-15

**Authors:** Kaveh Qaderi-Bagajan, Zahra Asgari, Matin Khanpayeh, Johanna Freund, Tobias Teismann, Lena Marie Hensel

**Affiliations:** 1 Department of Clinical and Health Psychology, Shahid Beheshti University, Tehran, Iran; 2 Department of Counseling, Faculty of Education and Psychology, University of Isfahan, Isfahan, Iran; 3 Department of Clinical Psychology, Kurdistan University of Medical Sciences, Sanandaj, Iran; 4 Lower Bavaria Medical Campus, Mainkofen District Hospital, Deggendorf, Germany; 5 Mental Health Research and Treatment Center, Ruhr-Universität Bochum, Bochum, Germany; 6 Deutsches Zentrum für Psychische Gesundheit (DZPG) (German Center for Mental Health), Bochum/Marburg, Germany

**Keywords:** ambivalence model, Iranian patients, qualitative, suicidality, suicide attempters

## Abstract

**Background:**

The Ambivalence Model of Suicidality (ABS) posits that suicidal experiences are often accompanied by internal conflicts between the desire to live and the urge to die. The model further conceptualizes the suicidal process as consisting of three phases: the uncertainty phase, the transition phase, and the action phase. This qualitative study explores the experiences of Iranian suicide attempters through the lens of the ABS to assess its cross-cultural validity and clinical relevance.

**Materials and methods:**

Semi-structured interviews with 15 individuals at Qods Psychiatric Hospital in Sanandaj, Iran, who had recently attempted suicide were conducted. The interviews examined emotional states, internal conflicts, external influences, and decision-making processes aiming to test whether the narratives corresponded to the three ABS phases. A deductive qualitative content analysis was applied: data were transcribed verbatim and coded according to predefined categories (Ambivalence, Transition, Action). Coding was conducted by one author and reviewed by a second author to ensure consistency with the theoretical framework. Representative quotations were extracted to illustrate alignment with the model.

**Results:**

Evidence for all three phases of the ABS was found. Participants’ narratives reflected each of the model’s predefined phases. Ambivalence prior to the suicide attempt was reported in 13 interviews, often accompanied by exhaustion. The transition phase frequently involved a sudden shift into acute suicidality, with some participants describing dissociation. Ambivalence often persisted into the action phase.

**Conclusion:**

Findings support the applicability of the ABS in the Iranian context, highlighting ambivalence as a fluctuating yet persistent state throughout the suicidal process and across cultures. Incorporating the ABS into clinical practice might enhance therapeutic approaches, supporting suicide prevention efforts.

## 1 Introduction

Ambivalence is considered a central construct for understanding suicidal crises (1, 2). In his memoir, the philosopher Clancy (3) states “For the suicidally inclined person, vacillation about whether one wants to live or die is the norm rather than the exception” (p. 41). In general, ambivalence refers to the simultaneous existence of positive and negative evaluations regarding a given person, an experience, or other (sets of) objects (cf. 4). The term suicidal ambivalence refers to the fact that wishes/reasons to die (e.g., “I am a burden and others would be better off if I were dead”) are experienced simultaneously with wishes/reasons to live (e.g., “I don’t want my husband to think I didn’t love him”) or to not die (e.g., “I’m afraid of the pain involved in killing oneself”). In an online study, 94% of suicidal individuals reported that they knew such an “internal suicide debate” (5). In studies on individuals who survived a suicide attempt, 41% to 50% furthermore stated that they felt ambivalent about dying by suicide at the time they attempted and 36% to 43% felt ambivalent about having survived a suicide attempt (6). However, in contrast to the significance and prevalence of suicidal ambivalence, the construct has not been (explicitly) considered in contemporary theoretical models for understanding suicidal crises e.g., (7, 8).

On this background, Teismann and colleagues (9) have recently introduced the Ambivalence Mode of Suicidality (ABS). The ABS model divides the suicidal process into three phases: an uncertainty phase, a transition phase and an action phase. The model proposes that most individuals are initially ambivalent about the possibility of suicide cf., (5); this ambivalence can be of varying strength (10) and it appears to fluctuate over time (11, 12). The model also states that ambivalence might be experienced as an exhausting and stressful state cf., (13, 14), that contributes to rumination, overarousal and emotional turmoil in suicidal individuals. In consequence persistent suicidal ambivalence might fuel back into suicidal ideation. This uncertainty phase is proposed to last between minutes, days, weeks or – in the case of persistent suicidal ideation – even years cf., (15).

The decision to die by suicide marks the progression from the uncertainty phase to the so called transition phase. The ABS states that it cannot be predicted whether and when suicidal individuals enter the transition phase cf., (16–18). With recourse to so-called dynamic systems approaches (19) (de Beurs et al., 2021), the ABS rather uses the term tipping points to characterize the sudden shift into a state of imminent suicide risk. According to the model, ambivalence does not have to be resolved in order to enter the transition phase; it is rather assumed that ambivalence gets pushed aside in these moments cf., (20). Still, entering the transition phase is supposed to be accompanied by a change in mental state: It is assumed that individuals show a strong “focus on death” (21) or enter a dissociative state of mind in the immediate run-up to a suicidal act (22); in consequence experiencing ambivalence is expected to not play a particular role in the transition phase, which becomes illustrated by another quote from (3). It was a dark night. There were no arguments for or against suicide in my mind. I just knew what I wanted to do, and this time I was going to do it”. However, ambivalence might be reinstated depending on situational cues (e.g. the telephone is ringing) or when suicide means are not readily available. The transition phase is proposed to last only for a few minutes or hours cf., (15, 23).

The enactment of suicidal behavior marks the progression from the transition phase to the action phase. The action phase and the transition phase are deemed to be closely interwoven, and the ABS assumes that the psychological state of suicidal individuals does not differ between the two phases. However, the model emphasizes that ambivalent experiences can become reactivated even after the initiation of a suicidal act: The experience of pain during a suicidal act or the emergence of anxiety after taking medication can call into question the decision to die by suicide and put those affected in a state of ambivalence again.

Taken together the ABS makes the assumption that ambivalence accompanies the whole suicidal process (in varying strength). Furthermore, the model emphasizes that a thorough examination of the ambivalence experienced by suicidal individuals is the key to understanding and treating an individual in a suicidal crisis (9). Still, it has to be noted that despite the widespread consensus that ambivalence is a core characteristic of suicidal individuals (1, 2), there is comparatively little high-quality empirical research on suicidal ambivalence (6). In view of this, various model descriptions of the ABS must be regarded as provisional.

Against this background, the aim of the current qualitative study was to employ a deductive, theory-driven design to explore whether the narratives of Iranian suicide attempters regarding the process leading up to their suicide attempt corresponded to the three phases (uncertainty, transition, and action) of the ABS. Rather than generating themes inductively, the aim of the present analysis was therefore focused on identifying text segments (in-)consistent with these predefined categories. To date, this represents the first qualitative study on the ABS.

## 2 Materials and methods

Reporting followed the Consolidated Criteria for Reporting Qualitative Studies (COREQ; 24; see **Supplementary Table 1**).

### 2.1 Participants and recruitment

Fifteen individuals (9 females, 6 males; aged 18-45; see **Table 1**) who had attempted suicide within the past three months were recruited from February 2025 to March 2025 at the Qods Psychiatric Hospital in Sanandaj, Iran. Participants were selected through purposive sampling and concurrently analyzed till data saturation. Inclusion criteria included a recent suicide attempt, sufficient cognitive capacity to participate, and the consent to be interviewed. Individuals who were deemed unable to participate safely in an interview due to acute psychiatric instability or cognitive impairment were not approached.

**Table 1:** Socio-demographic characteristics of the sample.

Participant	Age	Gender	ICD-10 diagnoses	Suicide attempt history (time/s)	Method of last suicide attempt
1	37	Male	F60.3	3	Cutting
2	33	Male	F60.3	1	Swallowing razorblade
3	22	Female	F42	2	Head banging
4	27	Female	F32.9	1	Overdose
5	19	Male	–	2	Hanging
6	24	Female	F32.9, F60.3	2	Cutting
7	36	Male	F31.9	1	Hanging
8	19	Female	F41.2	1	Jumping from a bridge
9	41	Female	F41.0, F40.10, F32.9	1	Overdose + Cutting
10	40	Female	F32.9	1	Rat poison ingestion
11	30	Male	F32.9	1	Hanging
12	38	Female	F32.9	1	Overdose
13	41	Female	F32.9	2	Bleach ingestion
14	33	Male	F19.10, F60.3	1	Overdose
15	19	Female	F32.9	1	Cutting

### 2.2 Data collection

Semi-structured interviews were conducted in Persian by trained clinicians, covering topics such as emotional states prior to the attempt, ambivalence, dissociation, decision to die, surviving the attempt. All interviews were conducted in-person, lasted approximately 60 minutes, and were audio-recorded with consent. The interview guide was created based on a description of the ABS (9). First, the aim of the research was explained, and participants’ questions were clarified. Next, the interview guide (see **Supplementary Material**) began with open-ended questions followed by trigger questions. Finally, additional probing questions were used to encourage elaboration and clarify participants’ responses.

### 2.3 Data analysis

English versions of the Persian originals were translated separately by two authors and then compared to ensure consistency. The interviews were then transcribed verbatim and analyzed using a deductive, theory-driven approach consistent with directed qualitative content analysis (24). The initial coding frame was derived *a priori* from the ABS by the fourth and the last author, and applied consistently throughout the analysis without modification. The coding system was hierarchically structured around the ABS’ three main phases (Uncertainty Phase, Transition Phase, Action Phase). Within the Uncertainty and Transition Phases, theory-based subcodes were applied (Ambivalence, Exhaustion caused by ambivalence, Sudden Decision to Die, Dissociation), whereas segments relating to the Action Phase were coded directly at the phase level. An additional category, “Post-attempt Phase”, was created to capture expressions of ambivalence following a suicide attempt. Beyond the predefined coding framework, no systematic inductive theme development was conducted. Thus, the final coding system consisted of six codes (see **Table 2**), applied to a total of 82 coded segments across the 15 interviews. A segment was defined as a meaningful unit of text representing a coherent idea relevant to the coding framework. For the manuscript, representative quotations were selected to illustrate central patterns and provide examples of the identified categories. All data were coded by the last author using MAXQDA 24.6, which facilitated systematic coding and data retrieval. Coding decisions were based on conceptual definitions and operational criteria derived from the ABS. For instance, segments were coded as “Ambivalence” if an internal conflict between the wish to live and the desire to die was described. The “Transition Phase” was assigned when participants reported a sudden shift towards acute suicidality or a narrowing of cognitive and emotional alternatives. To enhance reliability, coding decisions and category assignments were subsequently reviewed by the fourth author, providing an additional layer of verification and ensuring consistency with the theoretical framework. Reflexivity was addressed through collaborative discussion and critical reflection. The authors involved in the analysis had prior clinical and research experience in suicidology and were familiar with the ABS framework before conducting the analysis. Coding decisions and the interpretation of interviews were repeatedly discussed between authors, particularly in cases in which segments did not fit clearly within the predefined coding framework. During manuscript revision, additional attention was paid to relational, moral, and cultural aspects of ambivalence extending beyond the ABS model.

**Table 2:** Overview of codes, operational definitions, frequencies, and illustrative quotations.

Code	Operational definition	*n* Interviews	*n* Segments	Example quote
Ambivalence	Internal conflict between the desire to live and the desire to die prior to the suicide attempt	13	21	“It was like two people arguing inside my head.” (P13)
Exhaustion caused by ambivalence	Emotional and cognitive strain resulting from prolonged internal conflict	6	6	“The thoughts wouldn’t stop—they’d spin in my head for hours.” (P8)
Sudden Decision to Die	Rapid shift into acute suicidality and commitment to act	12	24	“The decision took seconds.” (P6)
Dissociation	Emotional numbing, detachment, or cognitive blankness before or during the attempt	5	5	“I felt nothing and thought of nothing.” (P9)
Action Phase	Description of ambivalence during the enactment of suicidal behavior	7	10	“Even at that moment, when I grabbed the bleach bottle, there was a struggle in my mind.” (P13)
Post-attempt Phase	Emotional reactions and ambivalence after surviving the suicide attempt	10	16	“I was sad I hadn’t died but also happy—mostly because of my kids.” (P9)

Segment frequencies represent the number of coded passages. Interview frequencies indicate the number of participants in whose narratives the respective code occurred.

The study was approved by the Ethics Committee of the research center in Tehran, Iran (IR.ATU.REC.1399.093). No one opted out, and several participants voiced gratitude for the chance to share their thoughts and feelings. We assigned code numbers to protect the participants’ true identities. Given the sensitive nature of the discussion, the interviewer concluded each interview by ensuring that the participant’s emotional well-being was stable.

## 3 Results

The participant narratives reflect some of the core components of the ABS. Below is a structured presentation of the codes and subcodes, with illustrative participant quotes. An overview of all (sub)themes, including their operational definitions, segment frequencies, and illustrative quotations, is presented in **Table 2**.

### 3.1 Uncertainty phase

All participants were able to name both reasons for dying and reasons for living or not dying in the time before their suicide attempt. Reasons not to die were cited by eleven participants (73.3%): Fear of pain and physical harm alongside the fear of committing a sin, were the most cited inhibiting factors. In some cases, religious practices such as praying, reading the Quran, or performing ablution rituals were described as attempts to cope with suicidal thoughts (e.g., P9, P12). Twelve out of 15 participants (80.0%) cited reasons for living, with concerns for friends, relatives, and children being the most frequently cited reason for continuing to live. The sense of responsibility for these significant others was often named as something that connected the participants to life (e.g., P4, P9, P11, P13). One participant described her children still needed her and stated that if she committed suicide her “son would fall apart” (P13), while another participant explained that caring for his elderly parents had repeatedly prevented him from acting on suicidal thoughts (P11). Close relationships thus emerged as both protective and distress-related factors as family and marital conflicts were consistently described as main reasons for dying across interviews (e.g., P1, P2, P3, P4). Furthermore, several interviews suggested that cultural and gender-related expectations contributed to the emergence of ambivalence. Female participants in particular described experiences of dependency, marital conflict, social shame, or fears regarding divorce and social exclusion (e.g., P6, P12, P13). One participant stated that “in my family, a divorced woman isn’t even considered a person” (P12), while another described feeling trapped in abusive relationships due to financial dependency and gendered role expectations (P6). Male participants, by contrast, more frequently linked suicidal thoughts to perceived failure to fulfil culturally valued roles such as being financially successful (P11) or the man their father expected them to be (P14). Concerns regarding social reputation and family honor were also repeatedly present (e.g., P7, P12, P13). Overall, these findings reveal ambivalence as being closely intertwined with interpersonal relationships and social roles.

Thirteen out of fifteen participants (86.7%) explicitly described the perceived nature of their suicidal ambivalence, frequently portraying it as an internal struggle: “It was like a fight in my head” (P5). In six out of 15 interviews (40.0%) symptoms of exhaustion caused by ambivalence were reported. For example, one participant (P8) describes the emotional exhaustion to the point of self-harm as follows: “The thoughts wouldn’t stop—they’d spin in my head for hours until I just got a headache and felt more confused.” (P8). However, the interviews did not reveal any explicit statements as to whether ambivalence itself contributed to an intensification of suicidal ideation. Furthermore, based on the interviews, no precise conclusions can be drawn about the duration of the uncertainty phase. While some participants described longstanding and recurring internal conflicts lasting months or years (e.g., P9, P13), others referred primarily to the immediate hours preceding the suicide attempt (e.g., P7, P11).

### 3.2 Transition phase

The decision to take one’s own life was described by almost all participants (*n* = 12; 80.0%) as a spontaneous and sudden decision: “I didn’t think much—I just suddenly decided to go to the bridge near our house” (P7). In various interviews, it was confirmed that ambivalence wasn’t resolved before the decision to die was made. For example, one participant (P9) states: “But that day, it was like a war in my head. I wanted to go home, but I couldn’t take it anymore. I cried a lot before I jumped. The decision kind of formed in my mind. It was like all the doubts went quiet—not resolved, just … silenced. I was still scared, though. Not much time passed between deciding and going to the bridge—maybe an hour or less.” In two interviews (13.3%), situational cues, such as “pills on the kitchen counter” (P4) were reported as relevant to the sudden decision. The vast majority of participants (*n* = 11; 73.3%) reported feeling anxious, sad, and stressed after deciding to die by suicide; only two participants reported a sense of relief. In five interviews (33.3%) evidence of dissociative experiences prior to or during the suicide attempt were revealed. For example: “I was dazed. I didn’t feel anything anymore” (P4); “At first, I felt this weird numbness.” (P8).

### 3.3 Action phase

All participants in the current study entered the action phase and enacted suicidal behavior. In seven interviews (46.7%) participants reported that ambivalence was still present during the suicide attempt. Participant 5 depicts this very clearly: “Yeah, I was scared—I don’t want to lie. I have nothing to lie about. When the rope tightens around your neck, it hurts a lot. I’ve never told anyone this before, but I was praying for someone to come help me.” The vast majority of participants (n = 12; 80.0%) were found by family members and taken to the hospital. Three individuals (20%) were found by outsiders (neighbors, motorists).

### 3.4 Post-attempt phase

In the aftermath of the suicide attempt, ten participants (66.7%) reported ongoing ambivalence. Post-attempt ambivalence was often closely tied to interpersonal relationships and ongoing responsibility toward significant others, while simultaneously involving feelings of relief, shame, and guilt (e.g., P9, P11, P13). Participant 13 describes the conflicting feelings as follows: “I mostly felt relieved that my children wouldn’t grow up without a mother. But I still feel very bad. Now, I even feel guiltier toward them. I feel like I am not a good mother at all. I wish the ground would open and swallow me” (P13). Three participants (20.0%) indicated that they were disappointed to have survived their suicide attempt and still wanted to die, while two participants (13.3%) said that after their suicide attempt, they realized they wanted to live.

## 4 Discussion

This qualitative study explored the applicability of the ABS among Iranian suicide attempters. The patient reports are largely consistent with the assumptions of the ABS: ambivalence plays a significant role in the run-up to a suicidal attempt, but ambivalence is also reported during and after a suicidal act cf., (6), ambivalence is experienced as stressful cf., (13) and is frequently not resolved before a suicide decision is made cf., (20); in many cases, the decision to die by suicide is made quickly and spontaneously cf., (23). Individual reports indicated that situational cues play a role in the suicide decision and that some affected persons experience dissociative symptoms before attempting suicide cf., (22). However, the interviews also made clear that suicidal crises have a highly individual character. Furthermore, not all narratives fully aligned with the assumptions of the ABS framework. For instance, the interviews did not reveal any explicit statements suggesting that ambivalence itself intensifies suicidal thoughts. Similarly, only a minority of participants reported experiencing significant exhaustion as a result of prolonged ambivalence. Furthermore, not all narratives fully aligned with the assumptions of the ABS framework. For instance, the interviews did not reveal any explicit statements suggesting that ambivalence itself intensifies suicidal thoughts. Similarly, only a minority of participants reported experiencing significant exhaustion as a result of prolonged ambivalence. Moreover, two participants (P1, P2) did not explicitly describe suicidal ambivalence. Nonetheless, in one of the cases fears regarding death as well as wishes “to have a family” were expressed (P1), and in the other case the participant stated that the suicide attempt was primarily intended to affect his wife: “I didn’t want to die. I wanted to scare her” (P2). These findings suggest that the role and subjective experience of ambivalence may vary across suicidal crises. It should also be noted that the ABS (9) and the current patient reports describe the process surrounding a suicide attempt and not the process surrounding a suicide. It cannot be ruled out that decisions leading to suicide are made less spontaneously. At the same time, some of the interviewees did use highly-lethal suicide methods that could have led to death (25). Taken together, these preliminary findings suggest that further systematic investigation of the ABS model appears worthwhile and support the idea that suicidal ambivalence is a cross-cultural phenomenon (26). In such studies, a more detailed interview method (15, 27) could be used to gain a better insight into the temporal processes of a suicidal crisis. In addition, more explicit questions should be asked about certain types of experience, such as dissociations and attentional fixation on suicide, but also of symptoms such as rumination and overarousal as a result of suicidal ambivalence. Future studies should also be evaluated inductively in order to gain an even deeper understanding and, to identify new relevant aspects for the further development of the ABS model.

From a clinical-therapeutic perspective, the findings underline the relevance of ambivalence-focused interventions. The literature on Motivational Interviewing with suicidal individuals (28–30) offers detailed instructions in this regard. The therapeutic goal of dealing with suicidal ambivalence is that suicidal individuals should be brought into (emotional) contact with reasons that conflict with the intention to die; they should experience how suicide conflicts with personally relevant goals and values. This is ultimately intended to ensure that suicidal ideators step back from immediate implementation intentions and become motivated to stay alive (for the time being) and seek further (therapeutic) help. However, the precise exploration of ambivalent attitudes does not only serve to motivate patients, but also forms the backbone of an individual case conceptualization and further treatment planning (9). With the aim to prevent suicidal individuals from entering the transition phase, strategies for dealing with strong suicidal impulses and urges should be implemented in crisis intervention and psychotherapy for suicidal patients: Means restriction counselling (31), safety planning or crisis response planning (32, 33), and (cognitive and behavioral) skills training (32, 34) are known to support suicidal individuals in establishing behavioral control when confronted with suicidal impulses. The relevance of situational trigger cues and immediately available suicide means also became evident in several participant narratives, for example when one participant stated: “My eyes fell on the pills on the kitchen counter” (P4).

### 4.1 Limitations

Several limitations should be considered when interpreting the present findings. First, the small hospital-based sample limits the generalizability of the current findings. Further research should examine the applicability of the ABS model using larger and age-, gender- and culturally diverse samples. Second, the interview guide was informed by the ABS framework and may therefore have prompted responses consistent with the model. Moreover, the predominantly deductive analytical approach may have increased the risk of confirmation bias and limited the identification of themes extending beyond the ABS framework. Although reflexive attention was paid to relational, moral, and cultural aspects of ambivalence during data interpretation, alternative thematic structures may have been overlooked. Consequently, the findings should be interpreted cautiously, and further studies using alternative methodological approaches are needed to examine the model’s explanatory value. This is all the more true given that the translation process from Persian to English may have resulted in subtle losses of meaning or cultural nuances. Third, although coding decisions were reviewed and discussed between two authors, the primary coding was conducted by a single researcher. Furthermore, no formal inter-rater reliability was calculated. Future studies could strengthen methodological rigor by involving multiple independent coders. Finally, while the present study provides qualitative support for the phases described in the ABS, it cannot draw conclusions about the effectiveness of the therapeutic strategies derived from the model. Further research is needed to examine their clinical utility.

### 4.2 Conclusion

The interviews conducted provide qualitative support for the central assumptions of the ABS model and underline the importance of suicidal ambivalence throughout the entire suicidal process. In light of the limited empirical literature on the ABS, the present findings offer encouraging preliminary evidence of the model’s applicability in an Iranian cultural context. Further research is needed to examine the role of ambivalence within the suicidal process and its relevance for suicide prevention and psychotherapy, using larger, culturally diverse samples and complementary study designs.

## Data Availability

The raw data supporting the conclusions of this article will be made available by the authors, without undue reservation.

## References

[1] Evans G Farberow NL . The Encyclopedia of Suicide. New York: Facts on File (1988).

[2] Shneidman ES . The Suicidal Mind. Oxford: Oxford University Press (1996).

[3] Martin C . How Not to Kill Yourself: Portrait of a Suicidal Mind. New York: Pantheon books (2023).

[4] Conner M Sparks P . Ambivalence and attitudes. Eur Rev Soc Psychol. (2002) 12:37–70. doi: 10.1002/0470013478.ch2 41531421

[5] Harris KM McLean JP Sheffield J Jobes D . The internal suicide debate hypothesis: exploring the life versus death struggle. Suicide Life Threat Behav. (2010) 40:181–92. doi: 10.1521/suli.2010.40.2.181 20465353

[6] Teismann T Siebert AM Forkmann T . Suicidal ambivalence: A scoping review. Suicide Life Threat Behav. (2024) 54:802–13. doi: 10.1111/sltb.13092 38709556

[7] Joiner T . Why People Die by Suicide. Boston: Harvard University Press (2005).

[8] O'Connor RC Kirtley OJ . The integrated motivational-volitional model of suicidal behaviour. Philos Trans R Soc Lond B Biol Sci. (2018) 373. doi: 10.1186/s12888-026-08049-2 30012735 PMC6053985

[9] Teismann T Britton PC Forkmann T . Ambivalence model of suicidality ABS-model: an orientation model for the treatment of suicidal individuals. Front Psychiatry. (2024) 15:1449565. doi: 10.4324/9781003616221-8 39676911 PMC11638166

[10] Brown GK Steer RA Henriques GR Beck AT . The internal struggle between the wish to die and the wish to live: a risk factor for suicide. Am J Psychiatry. (2005) 162:1977–9. doi: 10.1176/appi.ajp.162.10.1977 16199851

[11] Ernst M Gemke TJ Olivi LJ O'Connor RC . Ambulatory assessment of suicidal ambivalence: The temporal variability of the wish to live and the wish to die and their relevance in the concurrent and prospective prediction of suicidal desire. Suicide Life Threat Behav. (2024) 54:831–43. doi: 10.1111/sltb.13120 39096098

[12] Fartacek C Fartacek R Schiepek GK Sturm J Aichhorn W Plöderl M . Dynamic association between suicidal ambivalence and suicide risk among individuals with a history of suicide attempts. Suicide Life Threat Behav. (2024) 54:860–8. doi: 10.1111/sltb.13096 38837423

[13] Bergmans Y Gordon E Eynan R . Surviving moment to moment: The experience of living in a state of ambivalence for those with recurrent suicide attempts. Psychol Psychother. (2017) 90:633–48. doi: 10.1111/papt.12130 28497887

[14] van Harreveld F Rutjens BT Rotteveel M Nordgren LF van der Pligt J . Ambivalence and decisional conflict as a cause of psychological discomfort: Feeling tense before jumping off the fence. J Exp Soc Psychol. (2009) 45:167–73. doi: 10.1016/j.jesp.2008.08.015 38826717

[15] Xu I Millner AJ Fortgang RG Nock MK . Suicide decision-making: Differences in proximal considerations between individuals who aborted and attempted suicide. Suicide Life Threat Behav. (2024) 54:814–30. doi: 10.1111/sltb.13127 39221628

[16] Franklin JC Ribeiro JD Fox KR Bentley KH Kleiman EM Huang X . Risk factors for suicidal thoughts and behaviors: A meta-analysis of 50 years of research. Psychol Bull. (2017) 143:187–232. doi: 10.1037/bul0000084 27841450

[17] Hawton K Lascelles K Pitman A Gilbert S Silverman M . Assessment of suicide risk in mental health practice: shifting from prediction to therapeutic assessment, formulation, and risk management. Lancet Psychiatry. (2022) 9:922–8. doi: 10.1016/s2215-0366(22)00232-2 35952701

[18] Woodford R Spittal MJ Milner A McGill K Kapur N Pirkis J . Accuracy of Clinician Predictions of Future Self-Harm: A Systematic Review and Meta-Analysis of Predictive Studies. Suicide Life Threat Behav. (2019) 49:23–40. doi: 10.1111/sltb.12395 28972271

[19] de Beurs D Bockting C Kerkhof A Scheepers F O'Connor R Penninx B . A network perspective on suicidal behavior: Understanding suicidality as a complex system. Suicide Life Threat Behav. (2021) 51:115–26. doi: 10.1111/sltb.12676 33624872 PMC7986393

[20] Galasiński D Ziółkowska J . The end of ambivalence. A narrative perspective on ambivalence in the suicidal process. Suicide Life Threat Behav. (2024) 54:888–99. doi: 10.1111/sltb.12676 38847574

[21] Joiner T . The Varieties of Suicidal Experience. New York: New York University Press (2024).

[22] Michel K . The Suicidal Person. New York: Columbia University Press (2023).

[23] Paashaus L Forkmann T Glaesmer H Juckel G Rath D Schönfelder A . From decision to action: Suicidal history and time between decision to die and actual suicide attempt. Clin Psychol Psychother. (2021) 28:1427–34. doi: 10.1002/cpp.2580 33687121

[24] Hsieh H-F Shannon SE . Three approaches to qualitative content analysis. Qual Health Res. (2005) 15:1277–85. doi: 10.1177/1049732305276687 16204405

[25] Tong A Sainsbury P Craig J . Consolidated criteria for reporting qualitative research (COREQ): a 32-item checklist for interviews and focus groups. Int J Qual Health Care. (2007) 19:349–57. doi: 10.1093/intqhc/mzm042 17872937

[26] Ammerman BA Fahlgren MK Sorgi KM McCloskey MS . Differences in Suicidal Thoughts and Behaviors Among Three Racial Groups. Crisis. (2020) 41:172–8. doi: 10.1027/0227-5910/a000621 31512928

[27] Millner AJ Lee MD Nock MK . Describing and Measuring the Pathway to Suicide Attempts: A Preliminary Study. Suicide Life Threat Behav. (2017) 47:353–69. doi: 10.1111/sltb.12284 27477787

[28] Britton PC Williams GC Conner KR . Self-determination theory, motivational interviewing, and the treatment of clients with acute suicidal ideation. J Clin Psychol. (2008) 64:52–66. doi: 10.1002/jclp.20430 18161032

[29] Britton PC Patrick H Wenzel A Williams GC . Integrating Motivational Interviewing and Self Determination Theory with Cognitive Behavioral Therapy to Prevent Suicide. Cognit Behav Pract. (2011) 18:16–27. doi: 10.1016/j.cbpra.2009.06.004 27773985 PMC5070475

[30] Britton PC Bryan CJ Valenstein M . Motivational Interviewing for Means Restriction Counseling With Patients at Risk for Suicide. Cognit Behav Pract. (2016) 23:51–61. doi: 10.1016/j.cbpra.2014.09.004 38826717

[31] Bryan CJ Rudd MD . Brief Cognitive Behavioral Therapy for Suicide Prevention. New York: Guilford (2018).

[32] Stanley B Brown GK . Safety Planning Intervention: A Brief Intervention to Mitigate Suicide Risk. Cognit Behav Pract. (2012) 19:256–64. doi: 10.1016/j.cbpra.2011.01.001 38826717

[33] Linehan MM . Cognitive-Behavioral Treatment of Borderline Personality Disorder. New York: Guilford Press (1993).

[34] Cai Z Junus A Chang Q Yip PSF . The lethality of suicide methods: A systematic review and meta-analysis. J Affect Disord. (2022) 300:121–9. doi: 10.1016/j.jad.2021.12.054 34953923

