# Supplementary material for: Exploring the ambivalence model of suicidality among Iranian patients: a qualitative study with suicide attempters
Source: Front Psychiatry. 2026 Jul 15;17:1852185. doi: 10.3389/fpsyt.2026.1852185 (PMC13416669; doi:10.3389/fpsyt.2026.1852185)
Supplement: Supplementary file 1 [file SupplementaryFile1.docx]

| **Supplementary Material: Table 1**  Consolidated criteria for reporting qualitative studies (COREQ): 32-item checklist | | |
| --- | --- | --- |
| **No. Item** | **Guide questions/description** | **Reported** |
| **Domain 1: Research team and reﬂexivity** | | |
| *Personal Characteristics* | | |
| 1. Interviewer/ facilitator | Which author/s conducted the interview or focus group? | Interviews were conducted by trained clinical psychologists. |
| 2. Credentials | What were the researcher’s credentials? E.g. PhD, MD | All interviewers were clinical psychologists with more than five years of professional experience. |
| 3. Occupation | What was their occupation at the time of the study? | Clinical psychologists. |
| 4. Gender | Was the researcher male or female? | All interviewers were female. |
| 5. Experience and training | What experience or training did the researcher have? | Interviewers had prior experience working with patients with suicidal ideation or behaviors. |
| *Relationship with participants* | | |
| 6. Relationship established | Was a relationship established prior to study commencement? | No prior relationship established |
| 7. Participant knowledge of the interviewer | What did the participants know about the researcher? e.g. personal goals, reasons for doing the research | Participants were informed about the aim of the study (investigating the process leading up to a suicide attempt), the voluntary nature of participation, confidentiality procedures, and audio recording prior to the interview. |
| 8. Interviewer characteristics | What characteristics were reported about the interviewer/facilitator? e.g. Bias, assumptions, reasons and interests in the research topic | Interviews were conducted by trained clinicians experienced in working with suicidal patients. The interviewers were not directly involved in participants’ treatment. Their clinical background may have influenced the interaction; therefore, efforts were made to create a non-judgmental and supportive interview atmosphere. |

| **Supplementary Table 1**  COREQ (continued) | | |
| --- | --- | --- |
| **No. Item** | **Guide questions/description** | **Reported** |
| **Domain 2: study design** | | |
| *Theoretical framework* | | |
| 9. Methodological orientation and Theory | What methodological orientation was stated to underpin the study? e.g. grounded theory, discourse analysis, ethnography, phenomenology, content analysis | Deductive, theory-driven approach based on ABS |
| *Participant selection* | | |
| 10. Sampling | How were participants selected? e.g. purposive, convenience, consecutive, snowball | purposive |
| 11. Method of approach | How were participants approached? e.g. face-to-face, telephone, mail, email | face-to-face in hospital |
| 12. Sample size | How many participants were in the study? | 15 participants |
| 13. Non-participation | How many people refused to participate or dropped out? Reasons? | No participants dropped out after consenting. Information on initial refusals was not systematically recorded. |
| *Setting* |  |  |
| 14. Setting of data collection | Where was the data collected? e.g. home, clinic, workplace | Qods Psychiatric Hospital |
| 15. Presence of non-participants | Was anyone else present besides the participants and researchers? | No |
| 16. Description of sample | What are the important characteristics of the sample? e.g. demographic data, date | Fifteen individuals (9 females, 6 males; aged 18-45) |
| *Data collection* |  |  |
| 17. Interview guide | Were questions, prompts, guides provided by the authors? Was it pilot tested? | The interview Guide is given in the Appendix.  Not pilot-tested. |

| **Supplementary Table 1**  COREQ (continued) | | |
| --- | --- | --- |
| **No. Item** | **Guide questions/description** | **Reported** |
| 19. Audio/visual recording | Did the research use audio or visual recording to collect the data? | Audio recording |
| 20. Field notes | Were ﬁeld notes made during and/or after the interview or focus group? | Field notes were not systematically recorded. |
| 21. Duration | What was the duration of the interviews or focus group? | 60 minutes |
| 23. Transcripts returned | Were transcripts returned to participants for comment and/or correction? | Transcripts were not returned to participants for comment or correction. |
| **Domain 3: analysis and ﬁndings** | | |
| *Data analysis* |  |  |
| 24. Number of data coders | How many data coders coded the data? | 1 primary coder + 1 reviewer. |
| 25. Description of the coding tree | Did authors provide a description of the coding tree? | Yes (Figure 1) |
| 26. Derivation of themes | Were themes identiﬁed in advance or derived from the data? | Themes identified in advance (deductive). |
| 27. Software | What software, if applicable, was used to manage the data? | MAXQDA 24.6 |
| 28. Participant checking | Did participants provide feedback on the ﬁndings? | No. |
| *Reporting* |  |  |
| 29. Quotations presented | Were participant quotations presented to illustrate the themes/ﬁndings? Was each quotation identiﬁed (e.g. ID)? | Yes. |
| 30. Data and ﬁndings consistent | Was there consistency between the data presented and the ﬁndings? | Yes. |
| 31. Clarity of major themes | Were major themes clearly presented in the ﬁndings? | Yes (Uncertainty, Transition, Action) |
| 32. Clarity of minor themes | Is there a description of diverse cases or discussion of minor themes? | Yes (Exhaustion, Dissociation, Post-attempt) |
| Note. Tong A, Sainsbury P, Craig J. Consolidated criteria for reporting qualitative research (COREQ): a 32-item checklist for interviews and focus groups. International Journal for Quality in Health Care. 2007. Volume 19, Number 6: pp. 349 – 357. | | |

**Supplementary Material: Interview guide**

| Date |  |
| --- | --- |
| Interviewer |  |
| Participant | Code:  Age:  Gender: |
| Diagnoses  (if available) |  |
| Notes |  |

**Introduction**

*Greeting*

Good afternoon, Mrs/Mr XX

My name is XX and I work as a researcher at the University of Isfahan. We have made an appointment for the interview today. Thank you very much for your time.

*Presentation of the project*

I would first like to briefly introduce the project so that you know why we are conducting these interviews. In our study, we are investigating the process leading up to a suicide attempt. Above all, we want to find out more about the process leading up to a suicide attempt, how and when the decision to attempt suicide was made, what the exact course of the suicide attempt was like and how you felt after the suicide attempt. These are very intimate questions, so I would always ask you to decide for yourself whether you would like to answer my questions or not. Of course, you can also stop the interview at any time without having to justify yourself. Do you have any questions first?

*Duty of confidentiality*

Before we begin, a note on confidentiality: Everything you tell me is subject to confidentiality, with the exception of the other project staff. However, they are also bound to confidentiality.

*Sound recording*

We record all interviews using a smartphone. The interview is then transcribed, after which the audio track is deleted. If you disclose information about yourself during the interview, this will be deleted from the transcription to ensure anonymity. Is that okay with you?

**Interview**

1. You mentioned that you tried to take your own life once or several times. How often did you attempt suicide in your lifetime? Perhaps you could give me a brief overview: When did suicide attempts occur and what methods did you use? (Note: Write down the time and method of each single suicide attempt).

2. Let's take a closer look at the last suicide attempt. Perhaps you could tell me how it came about and what exactly you did?
(Let the participant talk openly: Make sure that information about the time of the suicide attempt, the method of the suicide attempt and any medical treatment following the suicide attempt is recorded).

3. What were the reasons for dying at that time? What was going on in your life that made you think about suicide?

4. And at the same time, were there also reasons for living? Were there doubts? Or were there obstacles on the way to suicide? Were there any fears about suicide? (*If no ambivalence is reported, ask again whether there was ambivalence weeks or month before the suic*ide attempt After all, your life has been difficult for some time, what had prevented you from making use of the possibility of taking your own life up to this point?)

5 (*If there was ambivalence, i.e. reasons for and against dying; otherwise continue with question 6)*: How did you experience this ambivalence? Were there rapid changes between the motivation to die and the motivation to life? Was the experience of ambivalence stressful for you? Did you constantly ruminate about reasons for and against living/dying? How long did the ambivalence last before you decided to take your own life?

6. How did the intention to kill yourself come about? (*If there was ambivalence, i.e. reasons for and against dying:* Did the ambivalence resolve or did you "push the ambivalence aside". Did you need to resolve the ambivalence in order to make a decision?)

7. When exactly did you make the decision to kill yourself? How did the decision come about? What happened to the ambivalence after you made the decision? How much time passed between the decision to die by suicide and acting on the decision? Please describe exactly what you did after the decision (describe all the preparations for the suicidal act in detail).

8. How would you describe your mental state in the time after you decided to die by suicide? How did you feel?

9. Did you feel fear or pain when you attempted suicide? Were you still ambivalent at the time?

10. What happened next? How did you manage to survive?

11 How did you feel after you realized that you had not died? Were you relieved? Were you disappointed? Were you ambivalent here too?

12. Is there anything else that is important for understanding the suicidal process?
